# *N*-(2-Chloro­phenyl­sulfon­yl)-2,2-dimethyl­propanamide

**DOI:** 10.1107/S1600536811017429

**Published:** 2011-05-14

**Authors:** K. Shakuntala, Sabine Foro, B. Thimme Gowda

**Affiliations:** a Department of Chemistry, Mangalore University, Mangalagangotri 574 199, Mangalore, India; b Institute of Materials Science, Darmstadt University of Technology, Petersenstrasse 23, D-64287 Darmstadt, Germany

## Abstract

In the title compound, C_11_H_14_ClNO_3_S, the C—S—N—C torsion angle is −61.69 (17)°. In the crystal, inversion dimers linked by pairs of N—H⋯O hydrogen bonds occur, generating *R*_2_^2^(8) loops.

## Related literature

For the sulfanilamide moiety in sulfonamide drugs, see: Maren (1976 ▶). For the ability of sulfonamides to form hydrogen bonds in the solid state, see: Yang & Guillory (1972 ▶). For hydrogen-bonding modes of sulfonamides, see: Adsmond & Grant (2001 ▶). For our study of the effect of substituents on the structures of *N*-(ar­yl)-methane­sulfonamides, see: Gowda *et al.* (2007 ▶). For related structures, see: Gowda *et al.* (2008 ▶); Shakuntala *et al.* (2011**a* ▶,b* ▶).
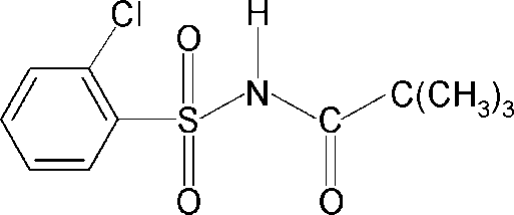


## Experimental

### 

#### Crystal data

C_11_H_14_ClNO_3_S
*M* *_r_* = 275.74
Triclinic, 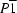

*a* = 8.785 (1) Å
*b* = 8.914 (1) Å
*c* = 9.313 (1) Å
α = 103.12 (1)°
β = 107.14 (1)°
γ = 94.20 (1)°
*V* = 670.98 (13) Å^3^
*Z* = 2
Mo *K*α radiation
μ = 0.44 mm^−1^
*T* = 293 K
0.44 × 0.40 × 0.38 mm

#### Data collection

Oxford Diffraction Xcalibur diffractometer with a Sapphire CCD detector
Absorption correction: multi-scan (*CrysAlis RED*; Oxford Diffraction, 2009 ▶) *T*_min_ = 0.831, *T*_max_ = 0.852
4355 measured reflections
2730 independent reflections
2321 reflections with *I* > 2σ(*I*)
*R*_int_ = 0.012

#### Refinement

*R*[*F* ^2^ > 2σ(*F* ^2^)] = 0.036
*wR*(*F* ^2^) = 0.107
*S* = 1.06
2730 reflections
158 parameters
1 restraint
H atoms treated by a mixture of independent and constrained refinement
Δρ_max_ = 0.26 e Å^−3^
Δρ_min_ = −0.26 e Å^−3^

### 

Data collection: *CrysAlis CCD* (Oxford Diffraction, 2009 ▶); cell refinement: *CrysAlis RED* (Oxford Diffraction, 2009 ▶); data reduction: *CrysAlis RED*; program(s) used to solve structure: *SHELXS97* (Sheldrick, 2008 ▶); program(s) used to refine structure: *SHELXL97* (Sheldrick, 2008 ▶); molecular graphics: *PLATON* (Spek, 2009 ▶); software used to prepare material for publication: *SHELXL97*.

## Supplementary Material

Crystal structure: contains datablocks I, global. DOI: 10.1107/S1600536811017429/tk2742sup1.cif

Structure factors: contains datablocks I. DOI: 10.1107/S1600536811017429/tk2742Isup2.hkl

Supplementary material file. DOI: 10.1107/S1600536811017429/tk2742Isup3.cml

Additional supplementary materials:  crystallographic information; 3D view; checkCIF report

## Figures and Tables

**Table 1:** Hydrogen-bond geometry (Å, °)

*D*—H⋯*A*	*D*—H	H⋯*A*	*D*⋯*A*	*D*—H⋯*A*
N1—H1*N*⋯O1^i^	0.83 (2)	2.22 (2)	3.042 (2)	178 (2)

Symmetry code: (i) 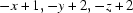.

**Fig. 1.**
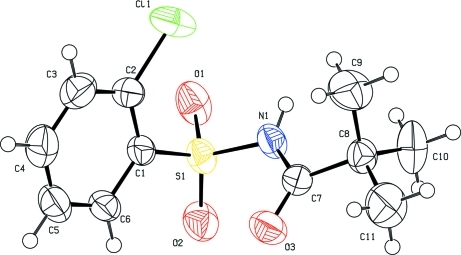
Molecular structure of the title compound, showing the atom-labelling scheme. Displacement ellipsoids are drawn at the 50% probability level.

**Fig. 2.**
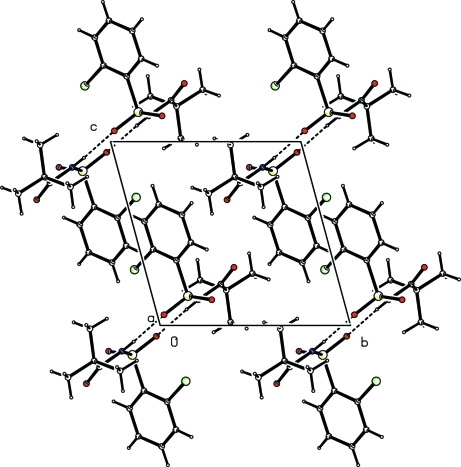
Molecular packing in the title compound. Hydrogen bonds are shown as dashed lines.

## supplementary crystallographic information

### Comment

The molecular structures of sulfonamide drugs contain the sulfanilamide moiety (Maren, 1976). The propensity for hydrogen bonding in the solid state, due to the presence of various hydrogen bond donors and acceptors, gives rise to polymorphism (Yang & Guillory, 1972). The hydrogen bonding preferences of sulfonamides has also been investigated (Adsmond & Grant, 2001). The nature and position of substituents in *N*-(aryl)sulfonoamides play a significant role on their crystal structures. As a part of a study of the substituent effects upon their crystal structures (Gowda *et al.*, 2007, 2008; Shakuntala *et al.*, 2011**a*,b*), in the present work, the crystal structure of *N*-(2-chlorophenylsulfonyl)-2,2,2-trimethylacetamide (I) has been determined. The N—H and C═O bonds in the SO_2_—NH—CO—C segment of (I) are *anti* to each other (Fig. 1), similar to that observed in *N*-(2-methylphenylsulfonyl)-2,2,2- trimethylacetamide (II) (Shakuntala *et al.*, 2011*a*), *N*-(phenylsulfonyl)-2,2,2-trimethylacetamide (III) (Gowda *et al.*, 2008) and *N*-(2-chlorophenylsulfonyl)-acetamide (IV) (Shakuntala *et al.*, 2011*b*). Further, the amide hydrogen is *syn* to the *ortho*-chloro group in the benzene ring.

The N—C bond in the C—SO_2_—NH—C segment has *gauche* torsion with respect to the S═O bonds. The molecule is twisted at the *S*- atom with a C—S—N—C torsion angle of -61.7 (2) °, compared to the values of -65.4 (2) ° in (II), -64.5 (3) ° in (III), and -71.7 (3) ° and 61.2 (3) ° in the two independent molecules of (IV).

In the crystal structure, the pairs of intermolecular N—H···O hydrogen bonds (Table 1) link the molecules into inversion-related dimers; a view of the crystal packing is shown in Fig. 2.

### Experimental

The title compound was prepared by refluxing 2-chlorobenzenesulfonamide (0.10 mole) with an excess of pivalyl chloride (0.20 mole) for about an hour on a water bath. The reaction mixture was cooled and poured into ice cold water. The resulting solid was separated, washed thoroughly with water and dissolved in warm dilute sodium hydrogen carbonate solution. The title compound was re-precipitated by acidifying the filtered solution with glacial acetic acid. It was filtered, dried and recrystallized from ethanol. Colorless prisms of the title compound used in X-ray diffraction studies were obtained from a slow evaporation of an ethanolic solution of the compound.

### Refinement

The H atom of the NH group was located in a difference map and later restrained to the distance N—H = 0.86 (2) Å The other H atoms were positioned with idealized geometry using a riding model with the aromatic C—H = 0.93 Å and methyl C—H = 0.96 Å. All H atoms were refined with isotropic displacement parameters (set to 1.2 times of the *U*_eq_ of the parent atom).

### Crystal data

**Table:**

C_11_H_14_ClNO_3_S	*Z* = 2
*M**_r_* = 275.74	*F*(000) = 288
Triclinic, *P*1	*D*_x_ = 1.365 Mg m^−^^3^
Hall symbol: -P 1	Mo *K*α radiation, λ = 0.71073 Å
*a* = 8.785 (1) Å	Cell parameters from 2522 reflections
*b* = 8.914 (1) Å	θ = 3.1–27.7°
*c* = 9.313 (1) Å	µ = 0.44 mm^−^^1^
α = 103.12 (1)°	*T* = 293 K
β = 107.14 (1)°	Prism, colourless
γ = 94.20 (1)°	0.44 × 0.40 × 0.38 mm
*V* = 670.98 (13) Å^3^	

### Data collection

**Table:**

Oxford Diffraction Xcalibur diffractometer with a Sapphire CCD detector	2730 independent reflections
Radiation source: fine-focus sealed tube	2321 reflections with *I* > 2σ(*I*)
graphite	*R*_int_ = 0.012
Rotation method data acquisition using ω and φ scans	θ_max_ = 26.4°, θ_min_ = 3.2°
Absorption correction: multi-scan (*CrysAlis RED*; Oxford Diffraction, 2009)	*h* = −10→10
*T*_min_ = 0.831, *T*_max_ = 0.852	*k* = −11→10
4355 measured reflections	*l* = −11→7

### Refinement

**Table:**

Refinement on *F*^2^	Secondary atom site location: difference Fourier map
Least-squares matrix: full	Hydrogen site location: inferred from neighbouring sites
*R*[*F*^2^ > 2σ(*F*^2^)] = 0.036	H atoms treated by a mixture of independent and constrained refinement
*wR*(*F*^2^) = 0.107	*w* = 1/[σ^2^(*F*_o_^2^) + (0.0533*P*)^2^ + 0.2343*P*] where *P* = (*F*_o_^2^ + 2*F*_c_^2^)/3
*S* = 1.06	(Δ/σ)_max_ = 0.004
2730 reflections	Δρ_max_ = 0.26 e Å^−^^3^
158 parameters	Δρ_min_ = −0.26 e Å^−^^3^
1 restraint	Extinction correction: *SHELXL97* (Sheldrick, 2008), Fc^*^=kFc[1+0.001xFc^2^λ^3^/sin(2θ)]^-1/4^
Primary atom site location: structure-invariant direct methods	Extinction coefficient: 0.129 (8)

### Special details

**Table:**

Experimental. CrysAlis RED (Oxford Diffraction, 2009) Empirical absorption correction using spherical harmonics, implemented in SCALE3 ABSPACK scaling algorithm.
Geometry. All e.s.d.'s (except the e.s.d. in the dihedral angle between two l.s. planes) are estimated using the full covariance matrix. The cell e.s.d.'s are taken into account individually in the estimation of e.s.d.'s in distances, angles and torsion angles; correlations between e.s.d.'s in cell parameters are only used when they are defined by crystal symmetry. An approximate (isotropic) treatment of cell e.s.d.'s is used for estimating e.s.d.'s involving l.s. planes.
Refinement. Refinement of *F*^2^ against ALL reflections. The weighted *R*-factor *wR* and goodness of fit *S* are based on *F*^2^, conventional *R*-factors *R* are based on *F*, with *F* set to zero for negative *F*^2^. The threshold expression of *F*^2^ > σ(*F*^2^) is used only for calculating *R*-factors(gt) *etc*. and is not relevant to the choice of reflections for refinement. *R*-factors based on *F*^2^ are statistically about twice as large as those based on *F*, and *R*- factors based on ALL data will be even larger.

### Fractional atomic coordinates and isotropic or equivalent isotropic displacement parameters (Å^2^)

**Table:**

	*x*	*y*	*z*	*U*_iso_*/*U*_eq_	
C1	0.1873 (2)	0.8220 (2)	0.6448 (2)	0.0421 (4)	
C2	0.2567 (2)	0.9285 (2)	0.5857 (2)	0.0506 (5)	
C3	0.1853 (3)	0.9346 (3)	0.4339 (3)	0.0635 (6)	
H3	0.2316	1.0059	0.3940	0.076*	
C4	0.0468 (3)	0.8358 (3)	0.3420 (3)	0.0666 (6)	
H4	−0.0012	0.8417	0.2405	0.080*	
C5	−0.0218 (2)	0.7287 (3)	0.3977 (3)	0.0603 (5)	
H5	−0.1153	0.6614	0.3341	0.072*	
C6	0.0485 (2)	0.7210 (2)	0.5489 (2)	0.0482 (4)	
H6	0.0026	0.6476	0.5868	0.058*	
C7	0.4718 (2)	0.6231 (2)	0.7753 (2)	0.0424 (4)	
C8	0.6484 (2)	0.6029 (2)	0.8078 (2)	0.0473 (4)	
C9	0.7309 (3)	0.7253 (3)	0.7535 (4)	0.0773 (7)	
H9A	0.7220	0.8274	0.8089	0.093*	
H9B	0.6802	0.7112	0.6439	0.093*	
H9C	0.8427	0.7144	0.7735	0.093*	
C10	0.7287 (3)	0.6256 (4)	0.9819 (3)	0.0817 (8)	
H10A	0.6750	0.5503	1.0166	0.098*	
H10B	0.7221	0.7287	1.0368	0.098*	
H10C	0.8398	0.6122	1.0018	0.098*	
C11	0.6588 (3)	0.4422 (3)	0.7175 (4)	0.0894 (9)	
H11A	0.6088	0.4308	0.6082	0.107*	
H11B	0.6044	0.3650	0.7496	0.107*	
H11C	0.7699	0.4287	0.7373	0.107*	
N1	0.44486 (18)	0.76248 (19)	0.86001 (19)	0.0483 (4)	
H1N	0.515 (2)	0.838 (2)	0.912 (2)	0.058*	
O1	0.29358 (17)	0.96456 (19)	0.94151 (18)	0.0656 (4)	
O2	0.16258 (17)	0.69277 (19)	0.86012 (18)	0.0635 (4)	
O3	0.36032 (17)	0.52985 (17)	0.68368 (18)	0.0614 (4)	
Cl1	0.43167 (8)	1.05545 (7)	0.69656 (9)	0.0820 (2)	
S1	0.26460 (5)	0.81187 (6)	0.83971 (5)	0.04740 (17)	

### Atomic displacement parameters (Å^2^)

**Table:**

	*U*^11^	*U*^22^	*U*^33^	*U*^12^	*U*^13^	*U*^23^
C1	0.0325 (8)	0.0377 (8)	0.0535 (10)	0.0082 (6)	0.0111 (7)	0.0092 (7)
C2	0.0415 (9)	0.0402 (9)	0.0667 (12)	0.0038 (7)	0.0138 (9)	0.0127 (8)
C3	0.0661 (14)	0.0577 (12)	0.0771 (15)	0.0134 (10)	0.0264 (12)	0.0318 (11)
C4	0.0639 (13)	0.0745 (15)	0.0584 (13)	0.0198 (12)	0.0086 (10)	0.0229 (11)
C5	0.0428 (10)	0.0636 (13)	0.0600 (12)	0.0055 (9)	0.0030 (9)	0.0062 (10)
C6	0.0353 (9)	0.0434 (9)	0.0613 (11)	0.0039 (7)	0.0132 (8)	0.0082 (8)
C7	0.0408 (9)	0.0427 (9)	0.0450 (9)	0.0069 (7)	0.0136 (7)	0.0142 (7)
C8	0.0415 (9)	0.0493 (10)	0.0518 (10)	0.0140 (8)	0.0151 (8)	0.0119 (8)
C9	0.0606 (14)	0.0830 (17)	0.104 (2)	0.0109 (12)	0.0483 (14)	0.0255 (15)
C10	0.0679 (15)	0.109 (2)	0.0668 (15)	0.0413 (15)	0.0077 (12)	0.0302 (14)
C11	0.0721 (16)	0.0639 (15)	0.118 (2)	0.0272 (13)	0.0259 (16)	−0.0034 (15)
N1	0.0327 (8)	0.0500 (9)	0.0538 (9)	0.0090 (6)	0.0079 (6)	0.0042 (7)
O1	0.0498 (8)	0.0707 (10)	0.0623 (9)	0.0196 (7)	0.0132 (7)	−0.0068 (7)
O2	0.0460 (8)	0.0832 (11)	0.0725 (10)	0.0109 (7)	0.0249 (7)	0.0343 (8)
O3	0.0470 (8)	0.0519 (8)	0.0717 (9)	−0.0013 (6)	0.0117 (7)	0.0019 (7)
Cl1	0.0639 (4)	0.0658 (4)	0.1008 (5)	−0.0238 (3)	0.0154 (3)	0.0167 (3)
S1	0.0350 (2)	0.0546 (3)	0.0507 (3)	0.01154 (19)	0.01324 (19)	0.0089 (2)

### Geometric parameters (Å, °)

**Table:**

C1—C6	1.389 (2)	C8—C9	1.524 (3)
C1—C2	1.388 (3)	C8—C10	1.524 (3)
C1—S1	1.7664 (19)	C9—H9A	0.9600
C2—C3	1.383 (3)	C9—H9B	0.9600
C2—Cl1	1.728 (2)	C9—H9C	0.9600
C3—C4	1.370 (3)	C10—H10A	0.9600
C3—H3	0.9300	C10—H10B	0.9600
C4—C5	1.368 (3)	C10—H10C	0.9600
C4—H4	0.9300	C11—H11A	0.9600
C5—C6	1.381 (3)	C11—H11B	0.9600
C5—H5	0.9300	C11—H11C	0.9600
C6—H6	0.9300	N1—S1	1.6434 (15)
C7—O3	1.202 (2)	N1—H1N	0.826 (16)
C7—N1	1.389 (2)	O1—S1	1.4274 (15)
C7—C8	1.523 (2)	O2—S1	1.4209 (16)
C8—C11	1.512 (3)		
			
C6—C1—C2	119.18 (18)	C8—C9—H9A	109.5
C6—C1—S1	117.88 (15)	C8—C9—H9B	109.5
C2—C1—S1	122.92 (14)	H9A—C9—H9B	109.5
C3—C2—C1	119.74 (18)	C8—C9—H9C	109.5
C3—C2—Cl1	118.34 (16)	H9A—C9—H9C	109.5
C1—C2—Cl1	121.92 (16)	H9B—C9—H9C	109.5
C4—C3—C2	120.2 (2)	C8—C10—H10A	109.5
C4—C3—H3	119.9	C8—C10—H10B	109.5
C2—C3—H3	119.9	H10A—C10—H10B	109.5
C5—C4—C3	120.8 (2)	C8—C10—H10C	109.5
C5—C4—H4	119.6	H10A—C10—H10C	109.5
C3—C4—H4	119.6	H10B—C10—H10C	109.5
C4—C5—C6	119.62 (19)	C8—C11—H11A	109.5
C4—C5—H5	120.2	C8—C11—H11B	109.5
C6—C5—H5	120.2	H11A—C11—H11B	109.5
C5—C6—C1	120.45 (19)	C8—C11—H11C	109.5
C5—C6—H6	119.8	H11A—C11—H11C	109.5
C1—C6—H6	119.8	H11B—C11—H11C	109.5
O3—C7—N1	120.27 (17)	C7—N1—S1	123.31 (13)
O3—C7—C8	124.89 (17)	C7—N1—H1N	125.0 (16)
N1—C7—C8	114.84 (15)	S1—N1—H1N	110.5 (16)
C11—C8—C7	109.01 (18)	O2—S1—O1	118.83 (10)
C11—C8—C9	109.4 (2)	O2—S1—N1	109.93 (9)
C7—C8—C9	108.39 (16)	O1—S1—N1	104.49 (9)
C11—C8—C10	110.8 (2)	O2—S1—C1	107.67 (9)
C7—C8—C10	109.76 (17)	O1—S1—C1	109.39 (9)
C9—C8—C10	109.4 (2)	N1—S1—C1	105.81 (8)
			
C6—C1—C2—C3	1.3 (3)	N1—C7—C8—C9	−65.3 (2)
S1—C1—C2—C3	−177.01 (16)	O3—C7—C8—C10	−126.5 (2)
C6—C1—C2—Cl1	−178.68 (14)	N1—C7—C8—C10	54.2 (2)
S1—C1—C2—Cl1	3.0 (2)	O3—C7—N1—S1	−1.5 (3)
C1—C2—C3—C4	−0.1 (3)	C8—C7—N1—S1	177.89 (13)
Cl1—C2—C3—C4	179.88 (18)	C7—N1—S1—O2	54.30 (18)
C2—C3—C4—C5	−0.9 (4)	C7—N1—S1—O1	−177.13 (15)
C3—C4—C5—C6	0.6 (3)	C7—N1—S1—C1	−61.69 (17)
C4—C5—C6—C1	0.6 (3)	C6—C1—S1—O2	0.55 (16)
C2—C1—C6—C5	−1.5 (3)	C2—C1—S1—O2	178.86 (15)
S1—C1—C6—C5	176.85 (15)	C6—C1—S1—O1	−129.89 (14)
O3—C7—C8—C11	−4.9 (3)	C2—C1—S1—O1	48.42 (17)
N1—C7—C8—C11	175.7 (2)	C6—C1—S1—N1	118.06 (15)
O3—C7—C8—C9	114.0 (2)	C2—C1—S1—N1	−63.63 (17)

### Hydrogen-bond geometry (Å, °)

**Table:**

*D*—H···*A*	*D*—H	H···*A*	*D*···*A*	*D*—H···*A*
N1—H1N···O1^i^	0.83 (2)	2.22 (2)	3.042 (2)	178 (2)

Symmetry codes: (i) −*x*+1, −*y*+2, −*z*+2.
